# The Birmingham Hospital Saturday Fund

**Published:** 1894-05-26

**Authors:** 


### THE INSTITUTIONAL WORKSHOP.

### HOSPITAL ADMINISTRATION.

### THE BIRMINGHAM HOSPITAL SATURDAY FUND.

[Previous articles will be found in THE HOSPITAL for May 12th, pages 127, 128, and May 19th, pages 149, 150.]

LAST week we published the reply of Mr. W. T. Smedley, the Honorary Secretary, to our remarks on the Birmingham Hospital Saturday Fund, which appeared in THE HOSPITAL of the 12th instant. In the brief footnote which we appended, by a printer's error it was stated that the registration fee of one shilling paid by an out-patient entails a loss of *two-and-sixpence*; it should have been *sixpence* per patient, on an average on the hospitals. A loss of sixpence, however, represents no less a sum than 33 per cent. of the estimated cost of each out-patient, and is, of course, an enormous loss in itself.

Turning now to Mr. Smedley's reply, every well-informed reader must perceive that it proves the correctness of the view which Mr. Burdett took of the position of affairs in Birmingham, and of the necessity for making a few friendly criticisms. Mr. Burdett gave emphatic testimony to the services rendered to the Birmingham Fund by the late Mr. Sampson Gamgee, Mr. G. F. Muntz, and Mr. George Dawson, and also by Mr. G. J. Johnson, the present Mayor. Mr. Smedley states that the credit thus apportioned was "heard with a certain amount of surprise." It is our duty, therefore, with the aid of documentary evidence in our possession, to state the facts which prove the value and character of the work done by these gentlemen in connection with the Birmingham Fund. Mr. George Dawson was a vitalising power in the life of the people of Birmingham during his ministry there, and he it was who presided at the Town Hall meeting on January 18th, 1869, the first birthday of the original Working Men's Committee, when he declared in language which many persons then thought to be figurative, but which has now become historical: "There is no reason why the working-classes should not turn charitable into co-operative institutions." Mr. George Dawson continued to support the movement with his pen and influence in the columns of the *Morning News*, a daily paper under his editorship, and at the meeting referred to the first Hospital Saturday collection was projected, although it did not actually take place until 1873. Speaking of the early days of this movement, Mr. Gamgee writes:—

> "Who was to tow the ship 'Self-Help' out of port? By common consent we turned to George Dawson, who was then in his prime, full of manly vigour, jolly and tender-hearted, brimful, nay, overflowing with human sympathies. Our ideas seemed to charm him, and he placed himself entirely at our disposal."

Of Mr. G. J. Johnson, the present Mayor of Birmingham, speaking of a highly critical episode in connection with the movement which occurred in November, 1872, Mr. Gamgee writes:—

> "Matters looked so awkward that I sought advice of a very influential and able friend, who had been instrumental in smoothing away other difficulties in our earlier progress. It was clear that the right policy was not to force the position, but to seek a compromise, for which the method was advised by Mr. G. J. Johnson. I hold in my hand the resolution, with his erasures and corrections, which resulted in an amalgamation of 'The Artisans' Medical Charities Fund' with the Hospital Saturday requisitionists to the Mayor, and the formation of a joint committee, with Mr. J. S. Wright chairman, myself honorary secretary, &c. In less than three months we banked over £4,700, the proceeds of our first Hospital Saturday, and Mr. G. F. Muntz, who had previously given £500 to the fund in aid of the Working Men's Fund for the extension of the Queen's Hospital, sent another £500 'as a mark of esteem for the noble effort and independent spirit of the working men.'"

We are confident that not only the working men of Birmingham, who were originally associated with this movement, and who now survive, but all working men will desire that full justice shall be done to every one of these pioneers, whose wise counsel, steady loyalty, and continuous help have done more probably than anything else to consolidate the Birmingham Hospital Saturday Fund, and to make it the success which it undoubtedly is to-day. We hope, therefore, that the "certain amount of surprise" referred to by Mr. Smedley was infinitesimal in quantity, and that the publication of these historical facts may prevent its reappearance in the future.

Mr. Smedley next contends that in 1878 "the original foundations of the Hospital Saturday movement in Birmingham were removed and others substituted," and "whatever success it has attained to-day has not been built on the foundations laid by Mr. Gamgee, but on foundations laid in direct opposition to his advice." Mr. Smedley declares the principles upon which the movement stands to-day to be: (1) "That it is the duty of men and women by systematic payments to make provision by which they can avail themselves of the advantages offered by the hospitals and dispensaries of the City;" and further (2) "to contribute towards the cost which the hospitals incur in relieving and healing those who have not the means of providing themselves with passports to the institutions," *i.e.*, it is the duty of all citizens to contribute towards the cost of the free work of the hospitals. It becomes a matter of historical interest and justice, in view of Mr. Smedley's challenge, to examine a little closely into the views Mr. Gamgee expressed in regard to the principles upon which the Fund should be administered. Mr. Smedley refers to the public address delivered by Mr. Gamgee on July 27th, 1882, that is, according to Mr. Smedley, four years after "the original foundations of the movement were removed and others substituted"—a copy of which lies before us as we write, and supplies precise evidence upon the points at issue. *De mortuis nihil nisi bonum* is a principle which governs the action of all fair-minded men, but the late Mr. Sampson Gamgee's work in connection with Hospital Saturday and the working-classes in Birmingham, like that of the late Mr. George Dawson, was too important and fruitful to need to be dealt with on any such sorry post mortem principle. We are full of astonishment that Mr. Smedley, with the knowledge of this address of Mr. Gamgee's which he intimates, could write as he has written in our issue of the 19th instant: "Building operations were commenced on altogether different lines in 1878." "Mr. Gamgee recognised this, and protested." A close and careful perusal of the address referred to has failed to bring to light any remarks of Mr. Gamgee's, "in which he strongly criticised the altered conditions under which the collection was conducted." Indeed, we believe a great service would be done, not only to the Hospital Saturday movement, but to the working-classes of Birmingham, if the Birmingham papers would republish Mr. Gamgee's address in full. It affords evidence that the principle upon which the Birmingham Hospital Saturday Fund stands to-day, as defined by Mr. Smedley, was the principle for which Mr. Gamgee contended. This address further shows that the success the Fund has attained to-day has been built on the foundations Mr. Gamgee laid, and not "on foundations laid in direct opposition to his advice." We are sorry to be compelled to meet Mr. Smedly with a direct negative upon these points, but as he has appealed unto Cæsar, there is no alternative left but to examine Cæsar's judgment.

First then, *on the duty of men and women making provision by systematic payment for the hospital relief they receive,* Mr. Gamgee writes: "As one means of promoting the Hospital Saturday collection, the penny-a-week system has been and is very useful. Nothing should be done to injure it—all it is posssible to supplement it. It is impossible to take too great care to safeguard the voluntary principle, which is the very essence of the movement. Every contributor will do well to bear in mind the frank and decisive words which Alderman Cooke addressed to his committee last month. He hoped their friends would make it known that the question of forcing had never entered the minds of the committee in any shape or form whatever, and they had always asked for the payments to be voluntary." Mr. Alderman Cooke has been the able chairman of the Hospital Saturday Fund Committee in Birmingham for very many years. Everybody who knows him will admit that he is a man of action, and that the policy of the Hospital Saturday Fund, under his chairmanship, would be largely a policy initiated and carried on by himself. What more convincing evidence of Mr. Gamgee's support of the principle upon which this branch of the Birmingham Fund is at present conducted could be adduced than his adoption of the penny-a-week plan of systematic payments, and of the views expressed by Alderman Cooke in regard to it?

Secondly, the principle upon which the Birmingham movement stands to-day includes, Mr. Smedley states, *the enforcement of the duty of each man and woman to contribute towards the cost of the free work of the hospitals.* On this point the Birmingham Fund has taken a new departure by fixing as a maximum the sum of £10,000 per annum to be handed over to the hospitals on Hospital Saturday, by establishing and conducting convalescent homes at the seaside, and by incorporating itself as a distinct society which appoints its own officers and manages its own affairs. All through his address Mr. Gamgee enforces the principle of working men representation on hospital committees, the necessity to keep their special work distinct and apart, and the importance of self-help. He further urges that "the Hospital Saturday Committee, enlarged if need be by wider working-class representation, is a body eminently fitted to be admitted to council." Was not the incorporation of the Birmingham Fund an enlargement in this sense, and was not Mr. Gamgee justified in his view, that

The contributions of a few more thousands annually might be stimulated by a modification of the existing plan, by grafting on it without uprooting it? Hospital Saturday has only as yet given an illustration of what may be done. The proceeds admit of being doubled with very little difficulty, and there is no valid reason why they should not be trebled.

After setting out the programme of what he called "Our Old Working Men's Hospital Charter," which was "as yet only partially accomplished," Mr. Gamgee goes on to enforce the importance that

The influence of the working classes should be felt in hospital administration in direct proportion to the growth of their hospital support.

Amongst the ways to accomplish this end Mr. Gamgee specifically mentions the following: The gift to some existing institution by the working classes of

"An accident ward, an operation theatre, a convalescent hall, or invalid baths, *an extension of the sanatorium at Blackwell,* and for the Hospital Saturday Fund to make arrangements on the co-operative principle to send some of Birmingham's sick toilers to the convalescent homes at Bath, Bournemouth, Buxton, Hastings, and the Isle of Wight." Further and strongly Mr. Gamgee writes: "Whatever method of collection for Hospital Saturday money is pursued, and I would oppose no method that would help in attaining the much needed success, I do hope that the plan will find increasing favour of making the day itself a day of labour sacrifice. I should like to see it become known, not only that the Birmingham workpeople on that particular day toiled an hour or more for their suffering fellows, but that they thus did better, because truer and more loving work, to honour and promote its practical benevolence."

To "make the day itself a day of labour sacrifice" is, of course, to contribute towards the cost of the free work of the hospitals.

It is true that Mr. Gamgee forcibly sets forth his objections to the registration fee, which we have already shown to be neither provident nor good business, and certainly in no sense charity. He declares, in fact, that the object of his address was:

Not to find fault, but so far as possible to assist; not to recall the painful part of lessons of experience, which every generous man knows how sweet it is to forget, but rather to cull from them suggestions for their practical use.

It is fair to conclude from these extracts that Mr. Smedley must have forgotten what this address contained, and on reperusal we believe he will be the first to admit the error into which he has inadvertently fallen, and to clear his mind of a misapprehension which does serious injustice to more than one of the great men now dead who have done good service for Birmingham and its institutions.

### The Present Position and Management.

Having thus disposed of the historical aspects of the questions at issue, we desire now to do justice to the present managers of the fund. It is universally recognised in Birmingham that Mr. Smedley is himself the life of the movement there at the present time. His untiring zeal, his energy, his devotion, the enthusiasm which he brings to bear upon the work, have all contributed to secure an army of men and women who take the deepest interest in the Hospital Saturday movement in Birmingham at the present time. We are thankful to realise the intensity of this interest, and to welcome the broadening and deepening of this movement now proceeding. The wisdom and judgment displayed by Mr. Alderman Cooke, the chairman of the committee for so many years, has materially added to the strength of the fund. Alderman Cooke is always a man of business, full of kindly feeling, and has the gift of winning his way to an extent possessed by few men. All honour to Mr. Alderman Cooke, Mr. Smedley, and their colleagues, who deserve well of the people of Birmingham. We are confident that the opening of the convalescent homes at Llandudno, and the decision to have a separate home for men and another for women is, wise, and well calculated to promote the growth and popularity of the fund. We can quite understand that the spirit which has been fostered amongst the workpeople by the belief that their gift is a free one, given to help those who are less fortunate than themselves, exercises a most powerful influence for good. For this reason it is essential to the very existence of the fund that this belief should be well founded. No one can, under present circumstances, prove this to be the case.

### The Future of the Birmingham Fund.

In such circumstances steps must be taken to obtain incontrovertible evidence on the point. In face of Mr. Smedley's figures, we feel that the claim put forward in regard to the amount of this free gift is, to say the least, founded on a misapprehension, and that the data upon which it is based require most careful examination at the hands of the Hospital Saturday Committee. We are forced to the conclusion, from the figures before us and from those supplied by Mr. Smedley himself, that, as a matter of fact, it is probable that the working classes in Birmingham receive more benefits from the hospitals than the sum raised on Hospital Saturday, plus the direct contributions to the hospitals by the working classes, would defray the cost of. In such circumstances, it is of the first importance that the Hospital Saturday Committee shall at once examine into the present system, and take steps to secure that in their next report accounts shall be given setting forth the actual sums of every kind given to the hospitals, apart from the Hospital Saturday Fund, by the working classes in Birmingham. We look forward to the time when the workshop collections will be so managed that each workshop will have its own doctor, who shall first be paid for the services which he renders to the sick workmen and their families who may require his help, and whose duty it will be to see all patients before they seek admission to the hospitals. Every hospital should issue books to the workshops, containing letters of introduction to the out-patient department, and every patient so introduced should be admitted to in or out patient treatment, according to the medical requirements of each case. In this way, and in this way alone, hospitals would be able to render to each workshop a statement showing the actual value of the relief given to its employés during the year. The employés would thus attain the happiness of feeling—to use Mr. Gamgee's words—"that a very large part of our population are not being manufactured into a race of beggars." Further, and of equal importance, full justice would be done to the medical profession. At the present time, we gather from Mr. Smedley's statement, that the utmost which is attempted is to purchase tickets for the employés of each workshop, and to provide them with a registration fee, which entails a loss of 33 per cent. upon the hospitals. This system leaves out of consideration the members of the families of the working classes who resort to the hospitals in large numbers, and the expense of whose treatment has not so far been taken into account. It is apparent that self-help includes provision by each head of a family, for wife and children, and hence the Birmingham Hospital Saturday Fund cannot yet claim that the balance found in the boxes on Hospital Saturday at present represents in any true sense a free gift from the working classes towards the free work of the hospitals. This contention, when all the facts are grasped, will not be disputed by any thoughtful person. It is a view which has been kept too long in the background, and we hope that the Birmingham Hospital Saturday Committee, as the responsible pioneers of the Hospital Saturday movement amongst the working classes, will now thoroughly probe it to the bottom. If they do so, we are confident that in the near future the action of the working classes through the Hospital Saturday Funds throughout the country will bring about a reform in hospital treatment honourable to themselves and worthy of a community which prides itself upon being in the van of human progress. This reform would be followed by the provision of an income large enough to defray the cost of every expenditure which the managers of the voluntary hospitals may find requisite to meet the medical needs of all members of the population to whom they may properly minister.

